# Variations in multimorbidity measurement in studies evaluating hospitalisation in older adults: a systematic review

**DOI:** 10.1186/s12877-025-05964-z

**Published:** 2025-11-07

**Authors:** Erika Aparecida Silveira, Andréa Toledo de Oliveira Rezende, Luciana Pereira Rodrigues, Bruno Pereira Nunes, Sandro Rogério Rodrigues Batista, Cesar de Oliveira

**Affiliations:** 1 Graduate Program in Health Sciences, School of Medicine, Federal University of Goiás, Goiânia, 74605-050 Brazil; 2 Department of Epidemiology & Public Health, University College London, London, WC1E 6BT UK; 3 Graduate Nursing Program, School of Nursing, Federal University of Pelotas, Pelotas, Brazil; 4 Department of Internal Medicine, School of Medicine, Federal University of Goiás, Goiânia, Brazil; 5 Postgraduate Program in Medical Sciences, Faculty of Medicine, University of Brasília, Brasília, Brazil

**Keywords:** Multimorbidity, Older adults, Hospitalisation, Systematic review

## Abstract

**Background:**

Multimorbidity is the coexistence of two or more chronic diseases, and in older adults is more common which can lead to increased rates of hospitalisation and health care. Therefore, this review aims to identify the variations in multimorbidity measurement in studies evaluating hospitalisation in older adults.

**Methods:**

A systematic review was conducted using a comprehensive database search in the PubMed, Embase, and Scopus databases, and included papers that used multimorbidity to evaluate hospitalisation in later life (PROSPERO register: CRD42021229328). Studies that employed multimorbidity measures with a simple count, weighted indices, Latent Class Analysis, Adjusted Clinical Groups System, Chronic Disease Score or Cumulative Index Illness Rating Scale were included. There was no restriction of language and year of publication of the studies included.

**Results:**

This review included 39 articles and was reported according to the PRISMA methodology. Analysing the variations in multimorbidity measurement related to hospitalisation in older adults we found a wide range of number of diseases, from 6 to 40, however, most of them utilised from 10 to 14 diseases. Regarding the data source, 56% of studies used self-reports, and 77% the disease count as a measure to assess multimorbidity.

**Conclusions:**

This review revealed enormous heterogeneity in the number and type of diseases and diverse methodological criteria applied in studies that assessed multimorbidity associated with hospitalisation in older adults. Furthermore, the disease group most used in the studies was those of the circulatory system and the five diseases frequently included were diabetes, hypertension, cancer, stroke, and coronary heart disease.

**Trial registration:**

PROSPERO: CRD42021229328.

## Introduction

Multimorbidity is commonly defined as the coexistence of two or more chronic diseases in the same individual [1]. It could include physical and mental health conditions as well as continuous disorders such as learning difficulties, complex symptoms like chronic pain, and substance misuse [2]. Increases in life expectancy have resulted in higher multimorbidity estimates in older adults. A study in Spain found that seven out of ten individuals aged 65 and older had two or more chronic diseases [3] while a systematic review showed that multimorbidity affects more than half of older adults worldwide [4]. Consequently, healthcare utilisation and hospitalisation levels have increased considerably [5].

There are several measures available to define multimorbidity that use different variables to create a score or index aiming to investigate diverse outcomes [6]. The most frequent approaches are the disease simple count, Charlson Comorbidity Index [7], Cumulative Illness Rating Scale [8], Adjusted Clinical Groups System [9], Cluster Analysis [10] and Latent Class Analysis [11], among others. Disease simple count is the most utilised method in multimorbidity research. It uses a diverse list of chronic conditions to identify how many of these conditions affect the same individual. This information is then analysed as a covariate to predict various outcomes such as mortality [12]. However, there is a great measurement of heterogeneity in the assessment of multimorbidity due to the lack of agreement on the number and type of diseases [13, 14] and lack of clarity of the selection criteria used to choose them in most studies [15]. Moreover, data sources vary i.e. self-reports, physician reports, medical records, or administrative data [17–19]. Generally, medical records and administrative data classify diseases according to the International Classification of Disease (ICD). However, the ICD is not applied in interviews and questionnaires, as diseases are presented to be intelligible to participants [16].

Currently, there is no consensus on the measures to define multimorbidity concerning methods, numbers and types of diseases, as well as data sources. Therefore, research on multimorbidity is challenging especially when a study aims to analyse its occurrence and association with various outcomes. This lack of consensus also hinders the comparison of results from different studies and the performance of a meta-analysis.

This systematic review is one of the earliest instances that we are aware of that identifies the diseases most frequently reported and evaluates the criteria used in multimorbidity research on hospitalisation in older adults. For this reason, this systematic review aims to verify similarities and disparities of the evidence on multimorbidity and hospitalisation focusing on the list and number of diseases, the existing assessment instruments, data source, and common diseases to evaluate multimorbidity among older adults. This age group was selected because multimorbidity affects over 50% of older adults globally and it has higher hospitalisation levels [18, 19]. This review also raises awareness of the main instruments used to study multimorbidity and hospitalisation in later life and the importance of having a consensus to define multimorbidity to facilitate both the understanding and comparison of outcomes. Thus, the purpose of this review is to identify how multimorbidity is measured in studies evaluating hospitalisation in older adults.

## Methods

### Protocol and registration

The reporting of this systematic review was guided by the Preferred Reporting Items for Systematic Reviews and Meta-Analyses (PRISMA) standards of quality [20]. The PECO framework [21] (Population, Exposition, Comparator and Outcome) was adopted as follows: “P” (older adults), “E” (multimorbidity), “C” (similarities and disparities in the studies) and “O” (hospitalisation). A protocol was developed and registered in PROSPERO (International Prospective Register of Systematic Reviews) (CRD42021229328). This registration number was used for this systematic review and another review entitled “Association between multimorbidity and hospitalisation in older adults: systematic review and meta-analysis” [22] that demonstrated an increased occurrence of hospitalizations and readmissions in older adults with multimorbidity. This review focused on the criteria used to measure multimorbidity in studies that connect multimorbidity to hospitalization. Further details may be found in the systematic review study protocol [23].

### Eligibility criteria and search strategy

The search was performed on PubMed, Embase and Scopus databases. In order to encompass all papers on this issue, we used mesh terms along with appropriate keywords such as multimorbidity, hospitalization, and older adults. There were no restrictions on the language or year of publication of the included research, and publications published from 15 December 2020 up to April 30, 2021 were examined.

The following inclusion criteria were applied in this review: (a) case–control, cohort, and cross-sectional studies; (b) multimorbidity definition as the presence of ≥ 2 and/or ≥ 3 chronic conditions in the same person; (c) multimorbidity measure using a simple count from various lists of chronic conditions; weighted indices as Charlson Index, as well as Cluster Analysis, Latent Class Analysis, Adjusted Clinical Groups System, Chronic Disease Score or Cumulative Index Illness Rating Scale, etc.; (d) outcomes that included hospitalisation, length of stay and readmission; (e) participants with age equal or over 60 years.

Exclusion criteria included review articles, ecological studies, case reports or case series, randomised clinical trials, and studies with missing, duplicate, or unavailable data even after contacting the authors. Studies that evaluated index disease (such as cancer, heart disease, or depression) or did not present the morbidities list considered in the study were also excluded. Therefore, studies focused on indigenous population were excluded as well as other age ranges, except if data related to older adults could be stratified.

### Review process

**Fig. 1:**
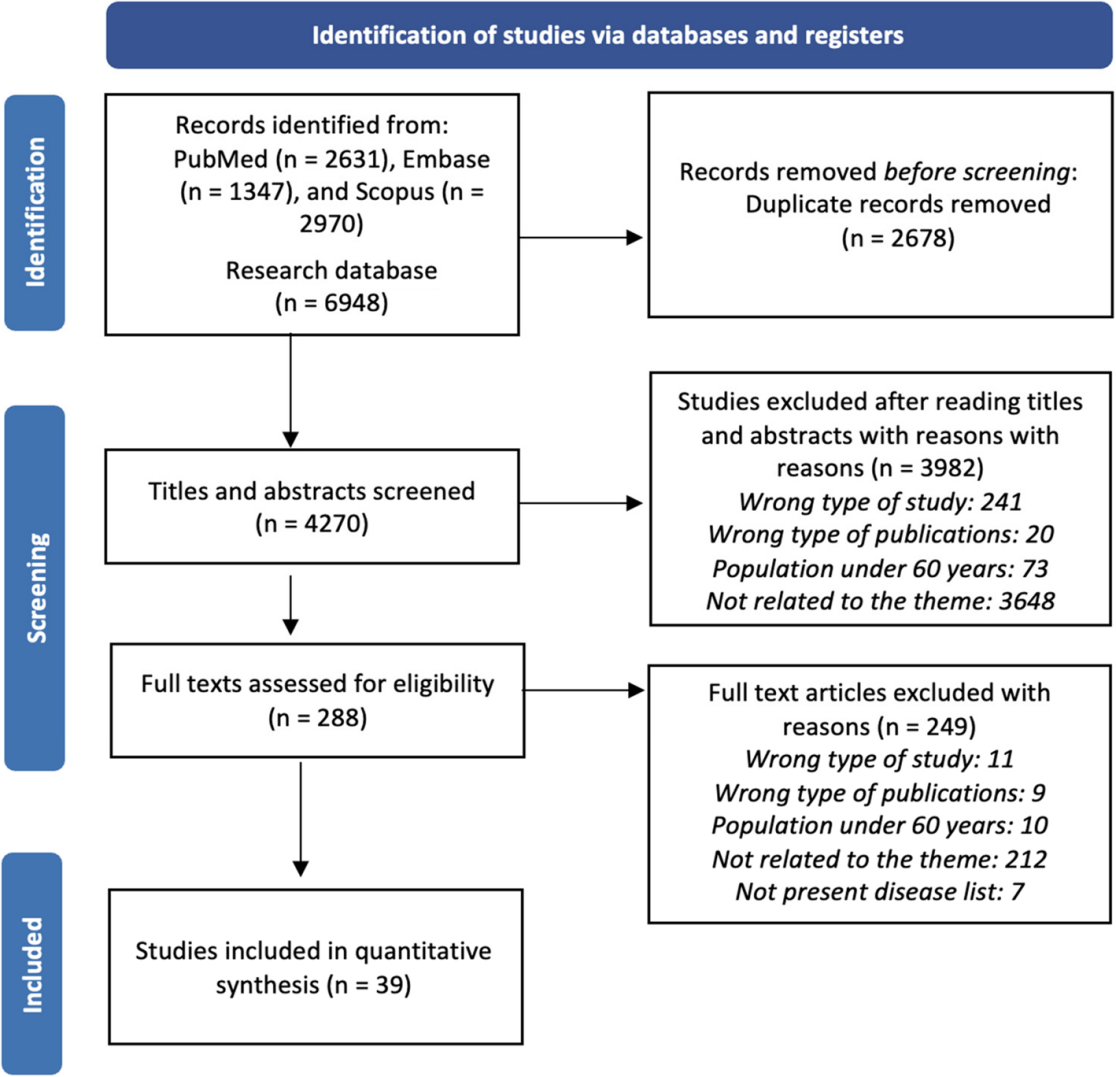
Flow diagram of articles selection stages

The search strategy was developed by two independent authors (ATOR and LPR) and duplicated studies were excluded using the Mendeley software. The authors read the titles and abstracts of all articles, those which were in accordance with the eligibility criteria were read in their entirety by the authors. The Rayyan software [24] was used in this process. Those studies considered eligible were included in the systematic review and available data extracted. If important data from some articles were unavailable, one of the authors contacted the respective researcher for clarification. Disagreements were discussed and resolved by a third reviewer (EAS). Figure 1 illustrates the PRISMA flowchart for the review process.

### Data extraction and quality assessment

Data were extracted from articles, then, they were inserted in a standardised form prepared by the authors including Author/Year, Disease List, Data Source/Instruments and Measure used to assess multimorbidity.

The Downs & Black Scale was used to evaluate the risk of bias during the study selection process. Studies were deemed to have a low probability of bias if their ratings were greater than 70%. The quality of the evidence was assessed using the Grading of Recommendations, Assessment, Development and Evaluations (GRADE). High quality (four full circles), moderate quality (three filled circles), low quality (two filled circles), and extremely low quality (one filled circle) were the grades assigned to the quality in each investigation. The evaluation of observational studies starts with two circles, and some factors—like the possibility of bias, imprecision, inconsistency, indirectness, and publication bias—may lower the quality of the evidence. On the other hand, if the effects are significant and all biases understate the effect or if there is a dose–response gradient, the quality of the evidence may improve (more full circles). The detailed process of evaluation quality of the articles for this systematic review may be found in previous publication [22].

## Results

In an initial search 6,948 articles were identified, out of which 4,270 articles remained after exclusion of duplicates. After applying the eligibility criteria, 288 were selected to be read in their entirety. Next, 249 articles were further excluded: 212 were not related to the theme, 11 applied other study designs, 10 used different populations, 9 comprehended other publication types, and 7 did not present disease list. Finally, 39 articles were included in the systematic review (Fig. 1). Considering the division by continent (Table 1), the following were identified: 10 studies in America, including 7 in North America [25–31] and 3 in South America [32–34]; 14 in Europe [35–48]; 11 in Asia [49–59]; 3 in Oceania [60–62]; and 1 used two different continents [63].

**Table 1:** List of diseases used in each study

Author/Year	Disease List	Data Source/Instruments	Measures used to assess multimorbidity	Setting
**AMERICA**				
**North America**				
Chamberlain et al. 2019 [25]	List of 18 CC: hypertension, congestive heart failure, coronary artery disease, cardiac arrhythmia, hyperlipidaemia, stroke, arthritis, asthma, cancer, chronic kidney disease, chronic obstructive pulmonary disease, dementia, depression, diabetes, osteoporosis, schizophrenia, substance abuse disorders (drug and alcohol) and, anxiety	Medical Reports/ Diagnostic codes	Diseases count	Community
Wagner et al.2019 [26]	List of 9 CC: malignant cancer/leukaemia, chronic pulmonary disease, coronary artery disease, congestive heart failure, severe chronic liver disease, chronic renal disease, dementia, diabetes with end organ damage, and peripheral vascular disease	Medical Reports	Diseases count based on ICD 9	Community
Gandhi et al. 2018 [27]	List of 15 CC: Alzheimer’s disease or dementia, asthma, atrial fibrillation, cancer, chronic kidney disease, chronic obstructive pulmonary disease, depression, diabetes, heart failure, high cholesterol, high blood pressure, ischemic heart disease, osteoporosis, arthritis, and stroke	Administrative data/ Medicare claims data	Diseases count based on ICD 9	Community
Ensrud et al. 2018 [28]	List of 31 CC: congestive heart failure, cardiac arrhythmias, valvular disease, pulmonary circulation disorders, peripheral vascular disorders, hypertension uncomplicated, hypertension complicated, paralysis, other neurological disorders, chronic pulmonary disease, diabetes uncomplicated, diabetes complicated, hypothyroidism, renal failure, liver disease, peptic ulcer disease excluding bleeding, AIDS, lymphoma, metastatic cancer, solid tumour without metastasis, rheumatoid arthritis/collagen vascular diseases, coagulopathy, obesity, weight loss, fluid and electrolyte disorders, blood loss anaemia, deficiency anaemias, alcohol abuse, drug abuse, psychoses, depression	Administrative data/ Medicare claims data	Diseases count based on ICD 9	Community
Gruneir et al. 2016 [29]	List of 16 CC: acute myocardial infarction, asthma, cancer, cardiac arrhythmia, chronic obstructive pulmonary disease, congestive heart failure, coronary syndrome, dementia, diabetes, hypertension, mood disorders, osteoarthritis, osteoporosis, renal failure, rheumatoid arthritis, or stroke	Administrative data/Physician billing codes	Diseases count based on prevalence and system burden	Community
Whitson et al. 2016 [30]	List of 13 CC: hypertension, arthritis (rheumatoid and/or non-rheumatoid), osteoporosis, diabetes mellitus, non-skin cancer, mental or psychiatric disorder, emphysema or chronic obstructive pulmonary disease, stroke, Alzheimer’s disease, Parkinson’s disease, heart arrhythmia, congestive heart failure, and coronary heart disease, which included myocardial infarction/heart attack, angina pectoris, or coronary heart disease	Self-reports	Diseases Count/Latent Class Analysis	Community
Lochner et al. 2013 [31]	List of 15 CC: arthritis, Alzheimer’s disease and related dementia, asthma, atrial fibrillation, cancer (breast, colorectal, lung, and prostate), chronic kidney disease, chronic obstructive pulmonary disease, depression, diabetes, heart failure, hyperlipidaemia, hypertension, ischemic heart disease, osteoporosis, and stroke	Administrative data/Medicare claims data	Diseases count	Clinics
**South America**				
Garcia-Ramirez et al. 2020 [32]	List of 7 CC: blood pressure, diabetes, osteoarthritis, ischemic heart disease, cerebrovascular disease, chronic respiratory disease, or cancer	Self-reports	Diseases count	Community
Nunes et al. 2017 [33]	List of 17 CC: systemic arterial hypertension, diabetes mellitus, pulmonary problems, heart disease, stroke, rheumatism/arthritis/osteoarthritis, spine problems, cancer, kidney failure; cognitive deficit evaluated by the Mini-Mental State Examination; Geriatric Depression Scale-evaluated depression; problems mentioned (urinary incontinence, amputation, visual or auditory problems, problem or difficulty chewing food, and falls	Self-reports	Diseases count	Community
Nunes et al. 2015 [34]	List of 17 CC: systemic arterial hypertension, diabetes mellitus, pulmonary problems, heart disease, stroke, rheumatism/arthritis/osteoarthritis, spine problems, cancer, kidney failure; cognitive deficit evaluated by the Mini-Mental State Examination; Geriatric Depression Scale-evaluated depression; problems mentioned (urinary incontinence, amputation, visual or auditory problems, problem or difficulty chewing food, and falls	Self-reports	Diseases count	Community
**EUROPE**				
Buja et al. 2020 [35]	List of 14 CC: cancer, congestive heart failure, ischemic heart disease, high blood pressure, cardiac arrhythmia, cerebrovascular disease, Alzheimer’s disease, depression, asthma/bronchitis, diabetes, chronic obstructive pulmonary disease, osteoporosis, hypothyroidism, and chronic renal disease	Administrative data/ Italian National Health System	ACG System	Clinics
Juul-Larsen et al. 2020 [36]	List of 22 CC: cancer, ulcers of the skin, dementia, depression, diabetes, disability, brain infarction/haemorrhage, hypertension, COPD/asthma, chronic kidney disease, osteoporosis/osteoarthrosis, Parkinson disease, arthritis, disorders of the eyes and ears, epilepsy, thyroid dysfunction, mental disorders due to alcohol, obesity, gastritis, cardiac disease, disorders of the lipoprotein metabolism and genitourinary diseases	Administrative data/ Danish National Patient Register	Latent Class Analysis	Community
Luben et al. 2020 [37]	List of 14 CC: myocardial infarction, congestive heart failure, peripheral vascular disease, cerebrovascular disease, dementia, chronic pulmonary disease, rheumatoid disease, peptic ulcer disease, liver disease, diabetes, hemiplegia or paraplegia, renal disease, cancer, and AIDS/HIV	Self-reports	Charlson Comorbidity Index	Community
Halonen et al. 2019 [38]	List of 9 CC: hypertension, heart disease, dementia, stroke, diabetes, arthritis, Parkinson’s disease, hip fracture and depression	Self-reports	Diseases count	Community
Jureviciene et al. 2018 [39]	List of 32 CC: cancer, anaemia, hypothyroidism, diabetes, obesity, dyslipidaemia, dementia, mental disorders, Parkinson's disease, multiple sclerosis, epilepsy, sleep apnoea, back pain, glaucoma, blindness, hearing loss, hypertension, ischaemic heart disease, arrhythmias, heart failure, intracranial bleeding, stroke, chronic obstructive pulmonary disease, asthma, inflammatory bowel disease, psoriasis, rheumatoid arthritis, gout, osteoarthritis, systemic lupus erythematosus, osteoporosis and renal failure	Administrative data/ Lithuanian National Health Insurance Fund	Diseases count based on ICD 10	Clinics/Hospital
Rodrigues et al. 2018 [40]	List of 12 CC: hypertension, diabetes, hypercholesterolemia, pulmonary disease, cardiac disease, gastrointestinal disease, neurologic disease, allergy, neoplastic disease, thyroid or parathyroid disease, hyperuricemia, rheumatic disease	Self-reports	Diseases count	Community
Olaya et al. 2017 [41]	List of 10 CC: depression, arthritis, asthma, COPD, angina, stroke, hypertension, diabetes, obesity, edentulism and cataract	Self-reports	Latent Class Analysis	Community
Collerton et al. 2016 [42]	List of 20 CC: hypertension, ischaemic heart disease, heart failure, atrial fibrillation or flutter, cerebrovascular disease, peripheral vascular disease, osteoarthritis, inflammatory arthritis, osteoporosis, chronic obstructive pulmonary disease, asthma, thyroid disease, diabetes mellitus, cancer, renal impairment, geriatric condition, urinary incontinence, falls, visual impairment, hearing impairment, cognitive impairment	Medical Reports	Cluster Analysis	Community
Hopman et al. 2015 [43]	List of 29 CC: hypertension, diabetes mellitus, asthma, coronary artery disease, COPD, chronic back or neck disorder, depression (and psychosis), osteoarthritis, cancer, cardiac dysrhythmia, visual disorder, osteoporosis, heart failure, migraine, anxiety disorder, stroke, neurasthenia/surmenage/burn-out, hearing disorder, rheumatoid arthritis, chronic alcohol abuse, epilepsy, heart valve disorder, dementia (incl. Alzheimer's disease), personality disorder, Parkinson's disease, schizophrenia, mental retardation, congenital cardiovascular anomaly, HIV/AIDS	Medical Reports	Cluster Analysis	Community
Navickas et al. 2015 [44]	List of 32 CC: cancer, anaemia, hypothyroidism, diabetes, obesity, dyslipidaemia, dementia, mental disorders, Parkinson’s disease, multiple sclerosis, epilepsy, sleep apnoea, back pain, glaucoma, blindness, hearing loss, hypertension, ischaemic heart disease, arrhythmias, heart failure, intracranial bleeding, stroke, chronic obstructive pulmonary disease, asthma, inflammatory bowel disease, psoriasis, rheumatoid arthritis, gout, osteoarthritis, systemic lupus erythematosus, osteoporosis and renal failure	Administrative data/ Lithuanian National Health Insurance Fund	Diseases Count based on ICD-10	Clinics/Hospital
Boeckxstae et al. 2015 [45]	List of 21 CC: hypertension, lipid disorder, angina pectoris, cardiomyopathy, myocardial infarct, transient ischemic attack, cerebrovascular accident, peripheral arterial disease, an episode of decompensated heart failure, an episode of atrial fibrillation, known valvular disease, thyroid disease, respiratory impairment [either asthma or chronic obstructive pulmonary disease, Parkinson’s disease, arthritis, osteoarthritis, documented osteoporosis, cancer, depression, renal insufficiency, locomotor sequelae of cerebrovascular accident, and diabetes	Self-reports	Diseases count, Charlson Comorbidity Index and Cumulative Illness Rating Scale	Community
Bähler et al. 2015 [46]	List of 22 CC: acid related disorders, bone diseases (osteoporosis), cancer, cardiovascular diseases (incl. hypertension), dementia, diabetes mellitus, epilepsy, glaucoma, gout/hyperuricemia, HIV, hyperlipidaemia, intestinal inflammatory diseases, iron deficiency anaemia, migraines, pain, Parkinson’s disease, psychological disorders (sleep disorder, depression), psychoses, respiratory illness (asthma, COPD),rheumatologic conditions, thyroid disorders, and tuberculosis	Administrative data/ Helsana Group claims data	Diseases count	Clinics/Hospital
Kadam et al. 2013 [47]	List of 6 CC: diabetes, hypertension, coronary heart disease, chronic obstructive pulmonary disease, congestive heart failure and chronic kidney disease	Administrative data/Not informed	Cluster Analysis	Clinics
Nägga et al. 2012 [48]	List of 14 CC: hypertension, hyperlipidaemia, urinary incontinence, arrhythmia, congestive heart failure, diabetes mellitus, affective disorders, stroke, cancer, asthma/chronic obstructive pulmonary disease, arthrosis, dementia, hypothyroidism and osteoporosis	Self-reports	Diseases Count based on ICD-10	Community
**ASIA**				
**China**				
Li et al. 2020 [49]	List of 14 CC: hypertension; dyslipidaemia; diabetes or high blood sugar; cancer; chronic lung disease; liver disease; heart disease; stroke; kidney disease; digestive disease; emotional, nervous, or psychiatric disease; memory-related disease; arthritis or rheumatism; and asthma	Self-reports	Diseases Count	Community
Zhao et al. 2020 [50]	List of 11 CC: hypertension, diabetes, dyslipidaemia, heart disease, stroke, cancer, chronic lung disease, digestive disease, liver disease, kidney disease, and arthritis	Self-reports	Diseases Count	Community
Lai et al. 2019 [51]	List of 40 CC: hypertension, depression, painful condition, asthma, coronary heart disease, treated dyspepsia, diabetes, thyroid disorders, rheumatoid arthritis, other inflammatory polyarthritis & systematic connective tissue disorders, hearing loss, chronic obstructive pulmonary disease, anxiety & other neurotic, stress related & somatoform disorders, irritable bowel syndrome, new diagnosis of cancer in last five years, alcohol problems, other psychoactive substance misuse, treated constipation, stroke & transient ischaemic attack, chronic kidney disease, diverticular disease of intestine, atrial fibrillation, peripheral vascular disease, heart failure, prostate disorders, glaucoma, epilepsy, dementia, schizophrenia (and related non-organic psychosis) or bipolar disorder, psoriasis or eczema, inflammatory bowel disease, migraine, blindness & low vision, chronic sinusitis, learning disability, anorexia or bulimia, bronchiectasis, Parkinson’s disease, multiple sclerosis, viral hepatitis, chronic liver disease	Medical Reports	Diseases count based on ICD 9	Hospital
Cheung et al. 2018 [52]	List of 7 CC: hypertension, diabetes mellitus, hypercholesterolemia, heart disease, stroke, chronic obstructive pulmonary disease, and renal disease	Self-reports	Diseases Count	Community
Wang et al. 2018 [53]	List of 17 CC: hypertension, chronic pain, diabetes mellitus, hyperlipidaemia, bone diseases, chronic gastrointestinal diseases, heart disease, gout, peripheral vascular disease, chronic kidney disease, spleen and gallbladder diseases, pulmonary disease, stroke, cancer, multiple sclerosis, dementia and mental disorder	Self-reports	Diseases Count	Community
**Other countries**				
Marthias et al. 2021 [54]	List of 14 CC: hypertension, diabetes, asthma, heart attack/coronary heart diseases, liver disease, stroke, cancer, arthritis/rheumatism, hypercholesterolaemia and depression/mental illness, prostate diseases, kidney diseases (excluding malignancy), digestive diseases and memory-related diseases	Self-reports	Diseases Count	Community
Kim et al. 2020 [55]	List of 28 CC: cancer, diabetes, thyroid disease, depression, otitis, vision problem, hypertension, dyslipidaemia, stroke, myocardial infarction or angina, haemorrhoids, ulcer, liver cirrhosis, temporomandibular joint dysfunction, hepatitis, arthritis, osteoporosis, backache, tuberculosis, asthma, chronic obstructive pulmonary disease, sinusitis, bronchiectasis, rhinitis, eczema, anaemia, kidney disease and urinary Incontinence	Self-reports	Diseases Count	Community
Pati et al. 2020 [56]	List of 21 CC: acid peptic disorder, hypertension, arthritis, chronic back pain, vision problem/blindness, diabetes, chronic lung disease, tuberculosis, deafness, thyroid disease, depression, heart disease, filariasis, eczema, dementia, psoriasis, kidney disease, alcohol disorder, stroke, hypotension, eczema, and psoriasis	Self-reports	Diseases Count	Community
Mitsutake et al. 2019 [57]	List of 21 CC: cancer, cerebrovascular accident, coronary heart disease, hypertension, peptic ulcer disease, dyslipidaemia, urologic disease, osteoarthritis, hyperuricemia, diabetes mellitus, arthritis, peripheral arterial disease, chronic obstructive pulmonary disease, asthma, anaemia; hypothyroidism; dementia; epilepsy; benign prostatic hypertrophy, Parkinson's disease and, chronic renal insufficiency	Administrative data/ Tokyo Extended Association of Medical Care System claims data	Diseases count based on ICD 10	Clinics/Hospital
Mini et al. 2017 [58]	List of 12 CC: rheumatism, osteoarthritis and osteoporosis, high-blood pressure, cataract, diabetes, lung disease, heart disease, paralysis, depression, Alzheimer’s disease, stroke (including cerebral embolism or thrombosis), dementia and, cancer	Self-reports	Diseases Count	Community
Picco et al. 2016 [59]	List of 10 CC: high blood pressure; heart trouble (including heart attack, angina, heart failure and valve disease); stroke; transient Ischemic attacks; diabetes; depression; arthritis or rheumatism; chronic obstructive pulmonary disease; breathlessness or asthma; and cancer	Self-reports	Diseases Count	Community
**OCEANIA**				
Shebeshi et al. 2020 [60]	List of 6 CC: hypertension, diabetes, heart disease, breast cancer, stroke and asthma	Administrative data/ Medicare claims data	Diseases Count	Community
Teh et al. 2018 [61]	List of 14 CC: coronary or peripheral artery disease; heart failure; stroke/ transient Ischemic attacks; any atrial fibrillation; diabetes; asthma or COPD; osteoporosis; osteoarthritis; rheumatoid arthritis; dementia; depression; thyroid disease; non-skin cancer and melanoma	Self-reports	Cluster Analysis	Community
Wister et al. 2016 [62]	List of 7 CC: arthritis/osteoporosis, asthma, blood pressure (hypertension), bronchitis/emphysema, cancer, diabetes, and heart disease	Self-reports	Diseases Count	Community
**OTHER CONTINENTS**				
Sum et al. 2020 [63]	List of 12 CC in Australia: arthritis/osteoporosis, asthma, cancer, chronic bronchitis/emphysema, type 1 diabetes, type 2 diabetes, depression, anxiety, other mental illness, heart disease, high blood pressure/hypertension, and any other serious circulatory condition List of 18 CC in Japan: heart disease, high blood pressure, hyperlipidaemia, cerebral/cerebrovascular accident, diabetes, chronic lung disease, asthma, liver disease, ulcer/other gastrointestinal disorder, joint disorder, osteoporosis, eye disease, ear disorder, bladder disorder, Parkinson's disease, depression/emotional disorder, dementia, and cancer	Self-reports	Diseases Count	Community

Regarding the variations in multimorbidity measurement we found a wide range of diseases used in the studies, the 10 most frequently diseases included were (Table 2): diabetes (in 38 of the 39 studies), hypertension (in 36 of the 39 studies), cancer (in 34 of the 39 studies), stroke (in 33 of the 39 studies), coronary heart disease (in 32 of the 39 studies), rheumatoid arthritis (in 31 of the 39 studies), asthma (in 30 of the 39 studies), Alzheimer’s disease (in 26 of the 39 studies), obstructive pulmonary disease (in 26 of the 39 studies), chronic kidney disease (in 24 of the 39 studies) (Table 2). However, some diseases were mentioned in only one article: allergy [46], anorexia [55], burn [39], cardiomyopathy [49], coagulopathy [30], congenital cardiovascular anomaly [39], constipation [55], digestive disease [52], disability [38], diverticular disease of the intestine [55], electrolyte disorders [30], endocrine disease [30], edentulism [47], filariasis [59], gastritis [38], drug abuse [30], hip fracture [44], hypotension [44], irritable bowel syndrome [55], learning disability [55], liver cirrhosis [59], mental retardation [39], organ damage [28], painful condition [55], psychoactive substance misuse [55], systemic lupus erythematosus [45], temporomandibular joint dysfunction [59], urologic disease [30], and weight loss [30]. Concerning the selection of diseases included in the studies about multimorbidity, 8 papers [28–30, 40, 42, 45, 55, 60] based their choice on ICD-9 or ICD-10, an International Code of Disease developed to promote the comparability of morbidity and mortality statistics between different countries [64].

**Table 2:** Rank of diseases considered in all the studies

	Lai et al. 2019 [51]	Jureviciene et al. 2018 [39]	Navickas et al. 2015 [44]	Ensrud et al. 2018 [28]	Hopman et al. 2015 [43]	Kim et al. 2020 [55]	Juul-Larsen et al.2020 [36]	Bähler et al. 2015 [46]	Pati et al. 2020 [56]	Mitsutake et al. 2019 [57]	Boeckxstae et al. 2015 [45]	Collerton et al. 2016 [42]	Sum et al. 2020 [63]	Chamberlain et al. 2019 [25]	Nunes et al. 2017 [33]	Nunes et al.2015 [34]	Wang et al. 2018 [53]	Gruneir et al. 2016 [29]	Gandhi et al. 2018 [27]	Lochner et al. 2013 [31]
**Number of diseases**	40	32	32	31	29	28	22	22	21	21	21	20	18	18	17	17	17	16	15	15
**Diseases**																				
diabetes	•	•	•	•	•	•	•	•	•	•	•		•	•	•	•	•	•	•	•
hypertension/high blood pressure	•	•	•	•	•	•	•	•	•	•	•	•	•	•	•	•	•	•	•	•
cancer	•	•	•	•	•	•	•	•		•	•	•	•	•	•	•	•	•	•	•
stroke/transient ischaemic attack/cerebrovascular accident/brain infarction/intracranial bleeding	•	•	•		•	•	•		•	•	•	•	•	•	•	•	•	•	•	•
coronary heart disease/ischaemic heart disease/coronary artery disease/cardiac disease/heart valve disorder/cardiovascular disease/valvular disease	•	•	•	•	•		•		•	•		•	•	•	•	•	•	•	•	•
rheumatoid arthritis/arthritis/rheumatic diseases/joint disorder/musculoskeletal disease	•	•	•	•	•	•	•	•	•	•	•	•	•	•	•	•		•	•	•
asthma/chronic respiratory disease/chronic lung disease/chronic pulmonary disease	•	•	•	•	•	•	•	•	•	•	•	•	•	•				•	•	•
Alzheimer’s disease/dementia/memory-related disease	•	•	•	•	•		•	•	•	•		•	•	•			•	•	•	•
obstructive pulmonary disease	•	•	•		•	•				•	•	•		•	•	•	•	•	•	•
chronic kidney disease/renal disease/chronic renal insufficiency/renal failure	•	•	•	•		•	•		•	•	•	•		•	•	•	•	•	•	•
depression	•			•	•	•	•	•	•		•		•	•	•	•			•	•
osteoporosis/osteoarthrosis/arthrosis/bone diseases		•	•		•	•	•	•			•	•	•	•			•	•	•	•
thyroid disorders/hypothyroidism	•	•	•	•		•	•	•	•	•	•	•								
heart failure/congestive heart failure	•	•	•	•	•	•					•								•	•
osteoarthritis		•	•		•						•	•			•	•		•		
Parkinson’s disease	•	•	•		•		•	•		•	•		•							
arrhythmias		•	•	•	•							•						•		
acid related disorders/acid peptic disorder/dyspepsia	•			•		•			•	•		•	•							
inflammatory bowel disease/chronic gastrointestinal diseases	•	•	•					•									•			
chronic liver disease	•			•									•							
peripheral vascular/peripheral arterial disease	•			•						•	•	•					•			
deafness/hearing impairment/hearing disorder	•	•	•		•				•						•	•				
epilepsy	•	•	•		•		•	•		•										
hyperlipidaemia								•			•		•	•			•			•
urinary incontinence/genitourinary diseases						•	•			•		•			•	•				
vision problems/visual disorder/eye disease					•	•	•					•	•		•	•				
schizophrenia/mental disorders/personality disorder	•	•	•		•								•				•			
anaemia		•	•	•		•		•		•										
atrial fibrillation	•										•	•							•	•
dyslipidaemia	•	•				•				•										
myocardial infarction/angina						•					•							•		
psoriasis/eczema/ulcus of the skin	•	•	•			•	•		•											
alcohol problems/alcohol abuse	•			•	•		•		•					•						
hypercholesterolemia/disorders of the lipoprotein metabolism							•												•	
anxiety/mood disorders/bipolar disorders	•				•								•	•				•		
hyperuricemia								•						•			•			•
stress related & somatoform disorders affective disorders/emotional disorder	•		•										•							
blindness/low vision	•	•	•						•											
glaucoma	•	•	•					•												
gout		•	•					•									•			
HIV				•	•			•												
obesity			•	•			•													
sleep apnoea		•	•												•	•				
back pain		•	•						•											
cognitive impairment												•			•	•				
falls												•			•	•				
geriatric condition												•			•	•				
migraine	•				•			•												
multiple sclerosis	•		•														•			
tuberculosis						•		•	•											
prostate disorders/benign prostatic hypertrophy	•									•										
bronchiectasis	•					•														
bronchitis/emphysema													•							
cataract																				
chronic sinusitis/rhinitis	•					•														
gallbladder diseases													•				•			
neurological disorders/neurologic disease				•																
otitis/ear disease						•							•							
paralysis/hemiplegia/paraplegia																				
psychosis				•				•												
spine problems															•	•				
viral hepatitis	•					•														
allergy																				
anorexia/bulimia	•																			
burn					•															
cardiomyopathy											•									
coagulopathy				•																
congenital cardiovascular anomaly					•															
constipation (treated)	•																			
digestive disease																				
disability							•													
diverticular disease of intestine	•																			
electrolyte disorders				•																
endocrine disease				•																
edentulism																				
filariasis									•											
gastritis							•													
drug abuse				•																
hip fracture																				
hypotension									•											
irritable bowel syndrome	•																			
learning disability	•																			
liver cirrhosis						•														
mental retardation					•															
neurologic disease																				
organ damage																				
painful condition	•																			
psychoactive substance misuse	•																			
systemic lupus erythematosus		•																		
temporomandibular joint dysfunction						•														
urologic disease										•										
weight loss				•																

	Buja et al. 2020 [35]	Luben et al. 2020 [37]	Teh et al. 2018 [61]	Nägga et al. 2012 [48]	Li et al. 2020 [49]	Marthias et al. 2021 [54]	Whitson et al. 2016 [30]	Rodrigues et al. 2018 [40]	Mini et al. 2017 [58]	Zhao et al. 2020 [50]	Olaya et al. 2017 [41]	Picco et al. 2016 [59]	Halonen et al. 2019 [38]	Wagner et al.2019 [26]	Garcia-Ramirez et al. 2020 [32]	Cheung et al. 2018 [52]	Wister et al. 2016 [62]	Shebeshi et al. 2020 [60]	Kadam et al. 2013 [47]	Number of studies that used this disease
**Number of diseases**	14	14	14	14	14	14	13	12	12	11	10	10	9	9	7	7	7	6	6	—
**Diseases**																				
diabetes	•	•	•	•	•	•	•	•	•	•	•	•	•	•	•	•	•	•	•	38
hypertension/high blood pressure	•			•	•	•	•	•	•	•	•	•	•		•	•	•	•	•	36
cancer	•	•	•	•	•	•	•	•	•	•		•		•	•		•	•		34
stroke/transient ischaemic attack/cerebrovascular accident/brain infarction/intracranial bleeding	•	•	•	•	•	•	•		•	•	•	•	•		•	•		•		33
coronary heart disease/ischaemic heart disease/coronary artery disease/cardiac disease/heart valve disorder/cardiovascular disease/valvular disease	•		•		•	•		•	•	•		•	•	•	•	•	•	•	•	32
rheumatoid arthritis/arthritis/rheumatic diseases/joint disorder/musculoskeletal disease		•	•		•	•	•	•	•	•	•	•	•				•			31
asthma/chronic respiratory disease/chronic lung disease/chronic pulmonary disease	•	•	•	•	•	•			•	•	•	•			•		•	•		30
Alzheimer’s disease/dementia/memory-related disease	•	•	•	•	•	•	•		•				•	•						26
obstructive pulmonary disease	•		•	•			•	•			•	•		•		•			•	25
chronic kidney disease/renal disease/chronic renal insufficiency/renal failure	•	•			•	•				•				•					•	24
depression	•		•			•			•		•	•	•							21
osteoporosis/osteoarthrosis/arthrosis/bone diseases	•		•	•			•		•											19
thyroid disorders/hypothyroidism	•		•	•				•												15
heart failure/congestive heart failure	•	•	•				•												•	14
osteoarthritis			•						•						•					11
Parkinson’s disease							•						•							11
arrhythmias	•			•			•													9
acid related disorders/acid peptic disorder/dyspepsia		•																		8
inflammatory bowel disease/chronic gastrointestinal diseases					•	•		•												8
chronic liver disease		•			•	•				•				•						8
peripheral vascular/peripheral arterial disease		•												•						8
deafness/hearing impairment/hearing disorder																				7
epilepsy																				7
hyperlipidaemia				•																7
urinary incontinence/genitourinary diseases				•																7
vision problems/visual disorder/eye disease																				7
schizophrenia/mental disorders/personality disorder							•													7
anaemia																				6
atrial fibrillation			•																	6
dyslipidaemia					•					•										6
myocardial infarction/angina		•					•				•									6
psoriasis/eczema/ulcus of the skin																				6
alcohol problems/alcohol abuse																				6
hypercholesterolemia/disorders of the lipoprotein metabolism						•		•								•				5
anxiety/mood disorders/bipolar disorders																				5
hyperuricemia				•																5
stress related & somatoform disorders affective disorders/emotional disorder					•															4
blindness/low vision																				4
glaucoma																				4
gout																				4
HIV		•																		4
obesity											•									4
sleep apnoea																				4
back pain																				3
cognitive impairment																				3
falls																				3
geriatric condition																				3
migraine																				3
multiple sclerosis																				3
tuberculosis																				3
prostate disorders/benign prostatic hypertrophy						•														3
bronchiectasis																				2
bronchitis/emphysema																	•			2
cataract									•		•									2
chronic sinusitis/rhinitis																				2
gallbladder diseases																				2
neurological disorders/neurologic disease								•												2
otitis/ear disease																				2
paralysis/hemiplegia/paraplegia		•							•											2
psychosis																				2
spine problems																				2
viral hepatitis																				2
allergy								•												1
anorexia/bulimia																				1
burn																				1
cardiomyopathy																				1
coagulopathy																				1
congenital cardiovascular anomaly																				1
constipation (treated)																				1
digestive disease										•										1
disability																				1
diverticular disease of intestine																				1
electrolyte disorders																				1
endocrine disease																				1
edentulism											•									1
filariasis																				1
gastritis																				1
drug abuse																				1
hip fracture													•							1
hypotension																				1
irritable bowel syndrome																				1
learning disability																				1
liver cirrhosis																				1
mental retardation																				1
neurologic disease								•												1
organ damage														•						1
painful condition																				1
psychoactive substance misuse																				1
systemic lupus erythematosus																				1
temporomandibular joint dysfunction																				1
urologic disease																				1
weight loss																				1

The diseases identified in the selected studies were further grouped into systems according to the ICD-10 (Table 3). The following systems were the most prevalent: circulatory, endocrine, nutritional, and metabolic, musculoskeletal system and connective tissue, and respiratory system. On the other hand, five diseases were less referenced: diseases of the eye and adnexa, certain infectious and parasitic diseases, diseases of the ear and mastoid process, diseases of the blood and blood-forming organs and certain disorders involving the immune mechanism, and diseases of the skin and subcutaneous tissue (Table 3).

**Table 3:** Rank of diseases grouped by system according to the ICD 10

GROUPS OF DISEASES	Number of studies that used each disease
**Circulatory system diseases**	
hypertension/high blood pressure	36
stroke/transient ischaemic attack/cerebrovascular accident/brain infarction/intracranial bleeding	33
coronary heart disease/ischaemic heart disease/coronary artery disease/cardiac disease/heart valve disorder/cardiovascular disease/valvular disease	32
heart failure/congestive heart failure	14
arrhythmias	9
peripheral vascular disease/peripheral arterial disease	8
atrial fibrillation	6
myocardial infarction/angina	6
hypotension	1
cardiomyopathy	1
TOTAL	146
**Endocrine, nutritional, and metabolic diseases**	
diabetes	38
thyroid disorders/hypothyroidism	15
hyperlipidaemia	7
dyslipidaemia	6
hyperuricemia	5
hypercholesterolemia/disorders of the lipoprotein metabolism	5
obesity	4
endocrine disease	1
weight loss	1
electrolyte disorders	1
TOTAL	83
**Musculoskeletal and connective tissue diseases**	
rheumatoid arthritis/arthritis/rheumatic diseases/joint disorder/musculoskeletal disease	31
osteoporosis/osteoarthrosis/arthrosis/bone diseases	19
osteoarthritis	11
back pain	3
painful condition	1
gout	4
spine problems	2
systemic lupus erythematosus	1
TOTAL	72
**Respiratory system diseases**	
asthma/chronic respiratory disease/chronic lung disease/chronic pulmonary disease	30
obstructive pulmonary disease	25
bronchiectasis	2
chronic sinusitis/rhinitis	2
bronchitis/emphysema	2
TOTAL	61
**Nervous system diseases**	
Alzheimer’s disease/dementia/memory-related disease	26
Parkinson’s disease	11
epilepsy	7
sleep apnoea	4
multiple sclerosis	3
migraine	3
paralysis/hemiplegia/paraplegia	2
neurologic disease	1
TOTAL	57
**Mental and behavioural disorders**	
depression	21
schizophrenia/mental disorders/personality disorder	7
alcohol problems/alcohol abuse	6
anxiety/mood disorders/bipolar disorder	5
stress related & somatoform disorders/affective disorders/emotional disorder	4
cognitive impairment	3
psychosis	2
anorexia/bulimia	1
drug abuse	1
learning disability	1
mental retardation	1
psychoactive substance misuse	1
TOTAL	53
**Genitourinary system diseases**	
chronic kidney disease/renal disease/renal failure/chronic renal insufficiency	24
urinary incontinence/genitourinary diseases	7
urologic disease	1
prostate disorders/benign prostatic hypertrophy	3
TOTAL	35
**Digestive system diseases**	
inflammatory bowel disease/chronic gastrointestinal diseases	8
chronic liver disease	8
acid related disorders/acid peptic disorder/dyspepsia	8
gallbladder diseases	2
constipation (treated)	1
diverticular disease of intestine	1
irritable bowel syndrome	1
liver cirrhosis	1
temporomandibular joint dysfunction	1
digestive disease	1
gastritis	1
ulcer	1
TOTAL	34
**Neoplasms**	
cancer	34
TOTAL	34
**Eye and adnexa diseases**	
vision problems/visual disorder/eye disease	7
blindness/low vision	4
glaucoma	4
cataract	2
TOTAL	17
**Infectious and parasitic diseases**	
HIV	4
tuberculosis	3
viral hepatitis	2
filariasis	1
TOTAL	10
**Ear and mastoid process diseases**	
deafness/hearing impairment/hearing disorder	7
otitis/ear disease	2
TOTAL	9
**Blood and blood-forming organs and immune system disorders**	
anaemia	6
coagulopathy	1
TOTAL	7
**Skin and subcutaneous tisseu diseases**	
psoriasis/eczema/ulcers of the skin	6
TOTAL	6
**Others**	
falls	3
hip fracture	1
allergy	1
burn	1
congenital cardiovascular anomaly	1
geriatric condition	3
disability	1
edentulism	1
organ damage	1
TOTAL	13

**Fig. 2:**
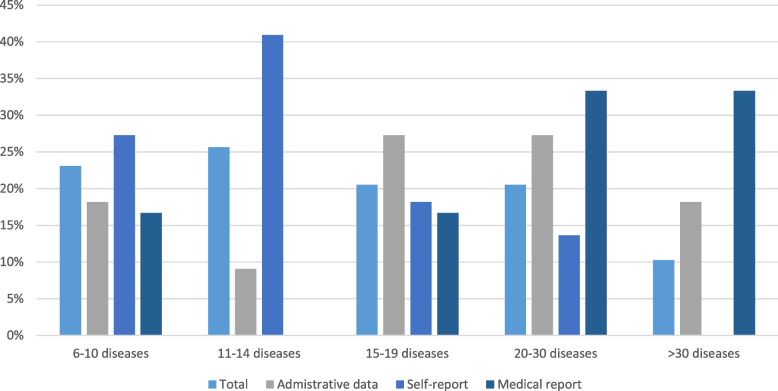
Number of diseases utilised in studies about multimorbidity measurement and hospitalisation in later life by data source type

The number of diseases included in the list of the studies ranged from 6 [41, 62] to 40 [55] as most of them used 11 to 14 diseases (26%) [32, 37, 42, 43, 46, 47, 51–53, 57, 61, 63], and 10% listed 30 or more [30, 40, 45, 55]. Regarding data sources to assess multimorbidity the following were employed: self-report (56%), administrative data (31%) and, medical report (13%). In the categories from 6 to 14 and 11 to 15 diseases, the data source frequently used was self-report, 15 to 19 was administrative data, 20 to 30 and > 30 disease was medical report (Fig. 2).

The measures adopted to assess multimorbidity found in review were Disease Count (30 articles), ACG system (1 article), Cluster Analysis (4 articles) [39, 41, 48, 63], Latent Class Analysis (3 articles) [32, 38, 47], Charlson Comorbidity Index (2 articles) [39, 45]), and Cumulative Illness Rating Scale (1 article) [49]. It is important to highlight that one article made use of three measures: Disease Count, Charlson Comorbidity Index, and Cumulative Illness Rating Scale [49].

## Discussion

To the best of our knowledge, this systematic review is among the first to identify the most often reported disorders and assess the standards applied in multimorbidity studies on hospitalization among older persons. Among the main findings, it is important to highlight the great heterogeneity in the type and number of diseases included in the articles and the methodological criteria applied in research focused on multimorbidity.

Our findings showed a wide differentiation considering the number of diseases, ranging from 6 to 40. However, most of the studies used 10 to 14 diseases to assess multimorbidity. Another study that examined the definition of multimorbidity in a population aged 18 and older found an extensive disparity in the number of diseases, ranging from 4 to 147 [65]. Concerning the disease type included in this review, 91 were identified, with some of them being mentioned in approximately 90% of the studies, as follows: diabetes, hypertension, cancer, and stroke. An overview on multimorbidity in the general population found that these four diseases were the most prevalent in the 51 studies included [66]. Furthermore, some diseases were counted as a single condition (i.e. cancer) while in other cases they were counted as specific individual conditions (i.e. stomach cancer). This finding is in accordance with a systematic review that assessed multimorbidity in the general population by using weight index and identified that the number and type of diseases varied greatly and most of the lists did not present any criteria in the selection of diseases [16].

Regarding the data source from which multimorbidity was assessed, this review identified that most studies used self-reports (56%), followed by administrative data (31%) and medical reports (13%). Some studies in the general population also identified that most data were self-reported [12], and it is important to note that in one of the articles, self-reports were present in 42% of the studies [67]. Therefore, it can be concluded that studies on the association between multimorbidity and hospitalisation were similar concerning the data source.

In most of the studies (77%) the disease count was the most used measure to assess multimorbidity related to hospitalisation, in contrast to just 2,6% that utilised the ACG System and Cumulative Illness Rating Scale. This is in line with a review on the population over 18 years old that emphasised the Disease Count as the most used method to measure multimorbidity due to its practicality, allowing comparison between studies since they present a similar diseases list [67]. This finding indicates similarity in the methodological characteristic between studies of multimorbidity and hospitalisation in the older adults.

Clinically, the key findings from this review support and highlight the importance of specifying how multimorbidity is defined, specifically the criteria applied when choosing the number and types of diseases included in the list. This is significant since there is currently no consensus on the disease lists, which is critical for developing models of care and recommendations to manage multimorbidity [68, 69]. Some consider chronic diseases, while others consider acute conditions, risk factors, and body systems, regardless of having the same outcome (hospitalisation) and population (older adults) [4, 66, 67].

In the public health’s perspective, our review highlighted the importance to clarify whether the criteria adopted for the choice of list of diseases was due to their prevalence in the study population, severity or continuous use of medication since these are aspects that can influence a study’s outcome. Thus, managers and policymakers may be encouraged to build, update, and adopt centred-person health systems, bringing this group's needs to the forefront of the health system [70].

Another important point raised by this review is that studies must clarify the reason for choosing measurement instruments to assess multimorbidity, since based on their objectives and consequent outcomes, it is necessary to adopt an appropriate measure that will allow to achieve accurate results. Considering all these aspects, it will be desirable to standardise the way of conducting research on multimorbidity and, therefore, help researchers in the future to investigate hospitalisation associated with multimorbidity in later life, and, ultimately, facilitate comparisons between studies. This will help health care professionals in the management of multimorbidity, since diverse expertise is required for effective treatment of this population [68, 71].

The main characteristic of this study is the broad database used to search for articles about the theme, being one American, another European, and a third one considered the largest database in scientific literature. Another point is that there were no restrictions regarding the language and year of publication, which enlarged the access to the articles related to the issue. The limitation of this review is that it may not have found all the studies that would meet the inclusion criteria due to the wide variety of terms used for multimorbidity, although we used a wide list of descriptors in the search strategy.

## Conclusion

This systematic review revealed diverse methodological criteria applied in the research focused on the studies of multimorbidity patterns. Evaluating these criteria, this review verified that in relation to similarities, the disease group most used in the studies was those of the circulatory system, and among the five diseases frequently included, the following are highlighted: diabetes, hypertension, cancer, stroke, and coronary heart disease. Regarding the number of diseases, most of the studies utilised 10 to 14 diseases to assess multimorbidity, and the most used measures and data sources were disease count and self-report.

Considering the differences, this systematic review revealed an enormous heterogeneity in the number and type of diseases included in the list of the studies that assessed multimorbidity associated with hospitalisation in older adults. Therefore, the results of this review support the importance of a methodological consensus in the studies on multimorbidity, especially related to creating a standardised list of diseases to obtain accurate and reproducible information since heterogeneity makes it difficult to compare studies.

## Data Availability

The data underlying this article will be shared on reasonable request to the corresponding author.

## Abbreviations

AIDS: Acquired immunodeficiency syndrome
CC: Chronic conditions
HIV: Human immunodeficiency virus
ICD-9: International Code of Disease version 9
ICD-10: International Code of Disease version 10
PRISMA: Preferred Reporting Items for Systematic Reviews and Meta-Analyses
